# Epigenetic regulator Cfp1 safeguards male meiotic progression by regulating meiotic gene expression

**DOI:** 10.1038/s12276-022-00813-0

**Published:** 2022-08-02

**Authors:** Byeong Seong Ki, Sung Han Shim, Chanhyeok Park, Hyunjin Yoo, Hyeonwoo La, Ok-Hee Lee, Youngjoo Kwon, David G. Skalnik, Yuki Okada, Ho-Geun Yoon, Jin-Hoi Kim, Kwonho Hong, Youngsok Choi

**Affiliations:** 1 Department of Biomedical Science, CHA University, Gyeonggi-do, 13488 Republic of Korea; 2 Department of Stem Cell and Regenerative Biotechnology, Humanized Pig Center, Konkuk University, Seoul, 05029 Republic of Korea; 3 College of Pharmacy, Graduate School of Pharmaceutical Sciences, Ewha Womans University, Seoul, 03760 Republic of Korea; 4 Department of Biology, School of Science, Indiana University-Purdue University Indianapolis, Indianapolis, IN 46202 USA; 5 Institute for Quantitative Biosciences, The University of Tokyo, Bunkyo, Tokyo 113-0032 Japan; 6 Department of Biochemistry and Molecular Biology, Yonsei University College of Medicine, Seoul, 03722 Republic of Korea

**Keywords:** Spermatogenesis, Epigenetic memory

## Abstract

Meiosis occurs specifically in germ cells to produce sperm and oocytes that are competent for sexual reproduction. Multiple factors are required for successful meiotic entry, progression, and termination. Among them, trimethylation of histone H3 on lysine 4 (H3K4me3), a mark of active transcription, has been implicated in spermatogenesis by forming double-strand breaks (DSBs). However, the role of H3K4me in transcriptional regulation during meiosis remains poorly understood. Here, we reveal that mouse CXXC finger protein 1 (Cfp1), a component of the H3K4 methyltransferase Setd1a/b, is dynamically expressed in differentiating male germ cells and safeguards meiosis by controlling gene expression. Genetic ablation of mouse CFP1 in male germ cells caused complete infertility with failure in prophase I of the 1st meiosis. Mechanistically, CFP1 binds to genes essential for spermatogenesis, and its loss leads to a reduction in H3K4me3 levels and gene expression. Importantly, CFP1 is highly enriched within the promoter/TSS of target genes to elevate H3K4me3 levels and gene expression at the pachytene stage of meiotic prophase I. The most enriched genes were associated with meiosis and homologous recombination during the differentiation of spermatocytes to round spermatids. Therefore, our study establishes a mechanistic link between CFP1-mediated transcriptional control and meiotic progression and might provide an unprecedented genetic basis for understanding human sterility.

## Male fertility: Regulatory protein’s role in making sperm

Details of the role of a protein in the development of sperm cells in mice could lead to new understanding of sterility in men. An international research team led by Youngsok Choi and Kwonho Hong at Konkuk University, Seoul, South Korea, investigated the role of protein Cfp1, which they found to be required for sperm formation in mice. The protein is a component of an enzyme complex that transfers methyl groups (CH_3_) onto other proteins involved in controlling gene activity. The researchers identified key aspects of the mechanism by which Cfp1 controls the activity of genes essential for sperm formation to proceed normally. Absence of Cfp1 specifically interferes with the process of meiosis, which generates sperm cells containing only one copy of each chromosome instead of the two copies found in other cells.

## Introduction

Spermatogenesis refers to the periodic differentiation of diploid spermatogonial stem cells (SSCs) into haploid spermatids, which engage in the germ cell-specific cell division process known as meiosis^1^. Therefore, meticulous regulation of meiosis guarantees the formation of sperm competent for sexual reproduction. Meiotic prophase involves double-strand breaks (DSBs) at preferred genome loci known as “hotspots”, formation of the synaptonemal complex, and homologous recombination via crossover at (pre)leptotene through pachytene stages^2,3^. Interestingly, trimethylation on histone H3K4 (H3K4me3) increases at the prophase stage of the 1st meiotic process and then decreases after diplotene cells and a brief reappearance in spermatids^4^. Studies have shown that H3K4me plays a role in the determination of hotspot locations^5,6^.

In mammals, PR/SET domain-containing protein 9 (Prdm9) marks meiotic recombination hotspots by modulating H3K4me3 enrichment^7–10^. Prdm9 disruption exhibits meiotic arrest with failure in synapse formation of homologous chromosomes and DSB formation^8,11–14^. However, the deletion of Prdm9 still shows recombination hotspots that were found at H3K4me3^15^. Interestingly, these H3K4me3 marks in the absence of Prdm9 are associated with ectopic recombination at the promoters and Prdm9-independent hotspots^15^.

It is generally appreciated that H3K4me results in transcriptional activation by allowing transcription factors to gain access to their target chromatin, and a subset of H3K4me2/3 modifications are enriched around active gene promoters, where RNA polymerase II (PolII) predominantly resides^16,17^. Genome-wide studies have shown that meiocytes are transcriptionally active and tightly regulate gene expression in response to cellular events, such as synapse formation and homologous recombination^18–20^. Notably, the mechanism of how the deposition of H3K4me3 at active gene promoters is differentiated from that which marks genomic hotspots for DSBs during meiosis has not yet been elucidated. Recently, CXXC finger protein 1 (CFP1) has been implicated as a key player in chromatin modification during meiosis^12,21^. Human CFP1 was initially reported to bind to nonmethylated CpG dinucleotide via the CXXC domain, and mutations in either the CXXC domain or methylated CpG would impair its binding affinity^22^. Later, it was shown that CFP1 is a crucial component of the H3K4 methyltransferase SET1 complex^23^ and facilitates H3K4me by recruiting the SET1 complex to the CpG region of active genes^24^. Mouse Cfp1 function is essential not only for embryogenesis^25^ but also for the development of various cells, including embryonic stem cells (ESCs)^26^, hematopoietic stem cells (HSCs)^27^, T-cell precursors^28^, myeloid cells^29^, and oocytes^30^. Loss of *Cfp1* in murine ESCs results in ectopic H3K4me, suggesting that Cfp1 is a critical regulator of H3K4me2/3 deposition at target gene promoters and activates gene expression^31^.

Alternatively, recent evidence suggests that Prdm9 might physically interact with Cfp1 via its KRAB domain in vitro^21^. Moreover, Prdm9 KO in spermatocytes did not affect global gene expression or enrichment of promoter-associated H3K4me3^14^. Therefore, given that distinct functions of Cfp1 have been suggested depending on its interacting enzymes and cell types (i.e., germ cell vs. somatic cells), we aimed to identify the role of Cfp1 in spermatogenesis in male gonads.

Herein, we showed that conditional deletion of Cfp1 in a mouse model resulted in infertility resulting from spermatogenetic arrest. We further demonstrated that Cfp1 is largely enriched at gene promoters and is a pivotal regulator of meiotic progression by controlling the transcription of genes essential for homologous recombination and synaptonemal complex formation.

## Materials and methods

### Animal care and generation of conditional knockout mice

All mice were obtained from the CHA University Animal Center (Seongnam, Republic of Korea). *Cfp1*^F/F^ transgenic mice were provided by Dr. Skalnik at Indiana University^27^. Tg (*Stra8*-icre*)* (Stock Number: 008208) mice were purchased from Jackson Laboratory and crossed with *Cfp1*^F/F^ mice to produce *Cfp1*^F/*+*^*;Stra8*-icre (control, CT) and *Cfp1*^F/F^*;Stra8*-icre (*Cfp1*^Stra8^) mice (Supplementary Fig. 1a, b). The primer sequences used for genotyping via PCR are listed in Supplementary Fig. 1d. Animal care and experimental procedures were performed according to the Guide for the Care and Use of Laboratory Animals and were approved by the Institutional Agricultural Animal Care and Use Committee of CHA University (IACUC No. 150015).

### Fertility test and histological analysis

The reproductive capabilities of CT and *Cfp1*^F/F^*;Stra8*-icre (*Cfp1*^Stra8^) male mice were tested, and fertile males were mated with wild-type females (C57BL/6 mice) over 6 months. All mating pairs of female mice were checked for vaginal plugs in the morning, and the number of litters was recorded. Testes were sectioned into 5-µm-thick slices and stained with hematoxylin and eosin (H&E) or subjected to immunofluorescence microscopy.

### RT–qPCR

Total RNA was extracted from the testes using an RNeasy total RNA isolation kit (Qiagen, Hilden, Germany) according to the manufacturer’s instructions. Total RNA (2 µg) was reverse-transcribed to synthesize complementary DNA (cDNA) using the SuperScript® III First-Strand Synthesis System (Life Technologies, Camarillo, CA, USA). QuantiTect SYBR Green PCR reagents (Qiagen) were used for RT–qPCR, and the results were evaluated with the iQ5™ Optical system software (Bio-Rad, Hercules, CA, USA). A list of PCR primer sequences is shown in Supplementary Table 1.

### TUNEL assays and immunostaining

Terminal deoxynucleotidyl transferase dUTP nick end labeling (TUNEL) assays were performed on paraffin-embedded tissue sections using an In Situ Cell Death Detection Kit (Sigma-Aldrich, St. Louis, MO, USA) according to the manufacturer’s instructions. For immunofluorescence, sections were incubated in blocking solution (PBST, 5% goat serum, 2% BSA) for 1 h at RT, incubated with primary antibodies overnight at 4 °C, incubated with fluorescent secondary antibodies for 2–3 h at room temperature, and counterstained with DAPI. Images were analyzed using a Zeiss LSM 750 confocal microscope (Carl Zeiss, Oberkochen, Germany). The antibodies used in this study are listed in Supplementary Table 2.

### Meiotic chromosome spread analysis

Meiotic cells of the P16 and P18 testes were isolated using a method adapted from previous studies^32,33^. Briefly, the decapsulated testes were incubated with collagenase (0.25 mg/ml) and deoxyribonuclease I (200 µg/ml) at 34 °C for 10 min. After incubation, dissociated testes tissues were diluted with DPBS, and the supernatant was removed. Tubules were incubated with trypsin (1 mg/ml) and deoxyribonuclease I (200 µg/ml) at 34 °C for 20 min. The digested tubules were filtered through a 70-µm and a 40-µm cell strainer and centrifuged at 1700 rpm for 10 min at 4 °C. The final pellet of meiotic cells was resuspended in 5 ml DPBS. Drops of hypotonic solution were then placed on the slides, and suspension cells were dropped. The spreads were fixed with fixative solution (2% formaldehyde, 0.02% SDS, pH 8.0). The chromosomes were dried for 30 min at RT and stained for immunofluorescence.

### Flow cytometry analysis of mouse male germ cells

Flow cytometry analysis of the germ cell population was performed as described in Vara et al.^34^. Briefly, seminiferous tubules were entangled in 1X PBS and incubated in 5 mg/ml collagenase type IV/PBS at 32 °C for 5 min. Germ cells were digested with 0.125% trypsin/50 U/ml DNase I at 32 °C for 8 min. Testes tissues were passed through a cell strainer (70 μm) and centrifuged. The cell pellet was resuspended in DMEM containing Hoechst 33342 (5 μg/ml) and DNase I (10 U) and incubated for 20 min at 32 °C. Then, the cells were resuspended, stained with propidium iodide (PI, 1 μg/ml), and immediately analyzed using CytoFLEX (Beckman Coulter, Brea, CA, USA).

### RNA preparation and microarray analysis

mRNA expression in whole testes from P14 CT or *Cfp1*^Stra8^ mice (*n* = 3/group) was compared using an RNeasy total RNA isolation kit (Qiagen) according to the manufacturer’s instructions. Biotinylated cRNA samples were prepared using 500 ng of total RNA according to the standard Affymetrix protocol (Affymetrix, Santa Clara, CA, USA). After fragmentation, 15 μg of RNA was hybridized at 45 °C for 16 h on a GeneChip Mouse Genome 430 2.0 Arrays. GeneChips were scanned using an Affymetrix GeneChip Scanner 3000 7G. The data were analyzed with RMA using Affymetrix default analysis settings and global scaling as the normalization method. The trimmed mean target intensity of each array was arbitrarily set to 100. The normalized and log-transformed intensity values were then analyzed using GeneSpring GX 12.5 (Agilent Technologies, Santa Clara, CA, USA). Hierarchical clustering data were used to cluster groups that behaved similarly across experiments using GeneSpring GX 12.5 (Agilent Technologies). The clustering algorithm was Euclidean distance and average linkage. The statistical significance of differentially expressed genes (DEGs) was determined when the difference in gene expression between *Cfp1*^Stra8^ and wild type had a *p* value ≤ 0.05.

### Spermatocyte isolation for ChIP-Seq analysis

Pooled mouse spermatocytes isolated from P21 mice were used for ChIP-Seq experiments. Seminiferous tubules from C57BL/6 and *Cfp1*^Stra8^ mice were removed, minced, and incubated with Krebs-Ringer bicarbonate media (EKRB) containing 0.5 mg/ml collagenase at 33 °C for 1 h. Dissociated seminiferous tubules were further digested with EKRB containing 0.5 mg/ml trypsin and 1 µg/ml DNase I at 33 °C for 15 min. Spermatocytes were then isolated using a method of sedimentation velocity at unit gravity at 5 °C. Cells suspended in EKRB/0.5% BSA were loaded onto a sedimentation chamber, and flow was applied (10 ml/min). Purified spermatocytes were subjected to lysis for ChIP-Seq analysis.

### ChIP-Seq and downstream analysis

The isolated spermatocytes were collected in a 1.5-ml tube, cross-linked with 1% formaldehyde (Sigma-Aldrich, F8775) for 10 min, and neutralized with 0.125 M glycine (Bio-Rad). Lysis buffer (5 mM PIPES pH 8.0, 85 mM KCl, 1% NP-40, 1 mM PMSF, 1X protease inhibitor cocktail) was added and incubated for 15 min at 4 °C. After centrifugation and removal of lysis buffer, 400 μl of nuclei lysis buffer [50 mM Tris-Cl pH 8.0, 10 mM EDTA pH 8.0, 1% SDS, 1 mM PMSF, 1X protease inhibitor cocktail (Roche, Mannheim, Germany)] was added and incubated at 4 °C for 30 min. Nuclei were sonicated using a probe-type sonicator (Qsonica, Newtown, CT, USA) at 4 °C for 20 cycles (30 s on and 30 s off at 40% amplitude) to produce approximately 300–400 bps of chromatin. After centrifugation, the supernatant was saved, and ice-cold IP dilution buffer (50 mM Tris-Cl (pH 7.5), 150 mM NaCl, 0.25% sodium deoxycholate, 1 mM EDTA (pH 8.0), 1% NP-40, 1 mM PMSF, 1X protease inhibitor cocktail) was added to the supernatant. Anti-Cfp1 (Bethyl Laboratories, Montgomery, TX, USA) antibody and a secondary antibody-conjugated Dynabead (Life Technologies) were added to the chromatin samples and incubated at 4 °C overnight. After phenol/chloroform extraction, immunoprecipitated DNA was eluted in TE buffer. For H3K4me3 ChIP-Seq, the ultralow input native ChIP (ULI-NChIP) method was used^35^. Briefly, cells were resuspended in 50 µl of nuclear isolation buffer (Sigma) on ice, treated with MNase Master Mix, and mixed by pipetting. The nuclear extracts were digested at 37 °C for 5 min and treated with 1% Triton-X100/1% deoxycholate solution to stop the reaction. Then, the chromatin was treated with complete IP buffer (20 mM Tris-HCl pH 8.0, 2 mM EDTA, 150 mM NaCl, 0.1% Triton X-100, 1X protease inhibitor cocktail, 1 mM PMSF) and incubated at 4 °C for 1 h. While incubating the chromatin with complete IP buffer, antibody [0.5–1 µg of H3K4me3 antibody (Active Motif)]-beads [prewashed protein A:protein G (1:1) Dynabeads (Life Technologies)] complex were prepared. Then, 150–200 µl of precleared chromatin was transferred to the antibody-bead complex and incubated at 4 °C overnight. The ChIP-Seq libraries were generated using a TruSeq Stranded IP sample preparation kit (Illumina, San Diego, CA, USA). ChIP-Seq raw data were aligned to the mm9 mouse genome using Bowtie2 (v2.2.9). ChIP-Seq peaks were called using MACS2 (v2.1.0). The following parameter was used for peak calling: “-B --nomodel -f BAM -g mm -p 1e-5”. Read per mean density after hierarchical clustering was generated using deepTools (v.3.1.3) or seqMINER (v1.3.3e). De novo motif finding was performed using Homer (v4.9.1).

### Reassessment of public datasets

Public RNA-Seq datasets with differentiating male germ cells (GSE35005) were downloaded and mapped to the mm9 mouse genome using the STAR tool (v2.5.2b). After mapping, fragments per kilobase million (FPKM) values were calculated using default options of Cuffnorm (v2.2.1, Cufflinks). ChIP-Seq datasets (GSE49624, GSE69946, GSE55471, GSE79227, and PRJNA281061 for H3K4me1/2/3 and GSE45441 for RNAP II in GS, PS, RS, and sperm) were downloaded. Reads per mean tag density (tag/50 bps) were plotted using seqMINER. RNA-Seq and ChIP-Seq data were visualized using Integrative Genomics Viewer (IGV). Functional annotation and enrichment analysis with DEGs were performed using DAVID (v6.8) and gene set enrichment analysis (v2.2.4, GSEA). The GO terms were visualized using the GOplot tool implemented in R (v3.3.2). The heatmap in RNA-Seq analysis was generated using the heatmap.2 tool (gplots package) in R. The prediction of disease caused by DEGs was performed with Ingenuity Pathway Analysis (IPA, Qiagen).

### Accession numbers

Microarray and ChIP-seq data have been deposited with the NCBI Gene Expression Omnibus (GEO) under GSE121240 (microarray) and GSE120994 (Cfp1 ChIP-Seq and H3K4me3 ChIP-Seq).

## Results

### Spermatogenetic arrest in *Cfp1* cKO leads to male infertility

**Fig. 1:**
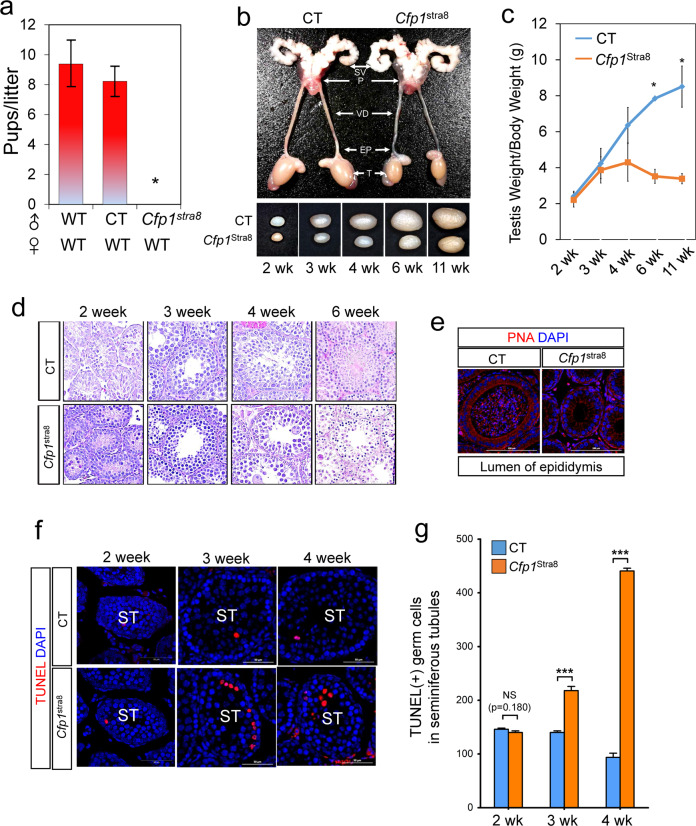
Male *Cfp1*^*Stra8*^ mice are infertile. **a** Fertility test in control (CT) and *Cfp1*^*Stra8*^ male mice by mating with wild-type (WT) females for 6 months. The numbers on the Y-axis represent the average pup size/litter. *p value < 0.001. **b** Top: Anatomical analysis of male reproductive organs of CT and *Cfp1Stra8* mice at 11 weeks of age. SV, seminal vesicle; P, prostate; VD, vas deferens; EP, epididymis; T, testis. Bottom: Morphological comparison. **c** Measurement of testes weight/body weight in CT (blue line) and *Cfp1*^*Stra8*^ (orange line) testes at various postnatal ages, from 2 weeks (2 weeks) to 11 weeks (11 weeks). *n* ≥ 4, *p value < 0.05. **d** H&E staining of cross sections of testes from 2-, 3-, 4-, and 6-week-old CT and *Cfp1*^*Stra8*^ male mice. Scale bars; 50 µm. **e** Immunostaining with PNA (red color) in the epididymis of CT and *Cfp1*^*Stra8*^ testes in 6-week-old mice. DNA was stained with DAPI (blue color). L, lumen. **f** TUNEL assay in CT and *Cfp1*^*Stra8*^ testes at 2, 3, and 4 weeks old. TUNEL-positive cells are shown in red, and DNA was stained with DAPI (blue color). ST, seminiferous tubule. **g** Quantification of TUNEL-positive cells in CT (blue bar) and *Cfp1*^*Stra8*^ testes (orange bar) (*n* ≥ 8). NS, no significant change; ***p value < 0.001.

To explore Cfp1 function in the testis, we next performed immunofluorescence in testicular seminiferous tubules containing germ cells in various stages of differentiation (Supplementary Fig. 1a). High levels of Cfp1 were expressed in Plzf(+) undifferentiated spermatogonia and Sertoli cells of postnatal day (P)7, P18, and P42 testes, whereas relatively low expression was observed in meiotic Sycp3(+) spermatocytes and PNA(+) spermatids. Sycp3(+)/Cfp1(+) spermatocytes were detected from P18 and remained in the seminiferous tubules of adult testes (P42). PNA(+)/Cfp1(+) spermatids were observed in the seminiferous tubules of P42 testes (Supplementary Fig. 1a). Given that Cfp1 remains high in undifferentiated spermatogonia during postnatal life, mice harboring *the Cfp1*-floxed allele (*Cfp*1^F/F^)^27^ were bred with Tg(*Stra8*-icre) mice to produce a male germ cell-specific Cfp1 cKO strain (hereafter referred to as *Cfp1*^Stra8^), as described in Supplementary Fig. 1b–e^36^. The Cre-mediated *Cfp1* deletion in testes was confirmed using PCR analysis (Supplementary Fig. 1d, e). We next determined fecundity in male *Cfp*1^Stra8^ mice by crossing them with wild-type (WT) female mice for over 6 months. *Cfp*1^Stra8^ males produced no pups, whereas *Cfp*1^F/+^; *Stra8*-icre males (control, CT) had fertility levels similar to those of WT mice (Fig. 1a).

We next examined morphological and histological features in *Cfp*1^Stra8^ testes and found that *Cfp*1^Stra8^ testes were hypoplastic compared to CT testes (Fig. 1b, c), whereas other cKO tissues, including seminal vesicle (SV), prostate (P), vas deferens (VD), and epididymis (EP) (Fig. 1b), were normal. Moreover, histological analysis of *Cfp*1^Stra8^ testes revealed a marked depletion of germ cells starting at three weeks of age (Fig. 1d). At 6 weeks of age, most seminiferous tubules in *Cfp*1^Stra8^ contained only spermatogonia, few spermatocytes and spermatids, and Sertoli cells (Fig. 1d). No mature sperm were detected in the epididymis of *Cfp*1^Stra8^ (Fig. 1e). Consistently, the number of TUNEL-positive cells was significantly increased in *Cfp*1^Stra8^ testes compared to that of CTs from 3 weeks of age (Fig. 1f, g). These findings indicate that deficiency of Cfp1 in male germ cells results in abnormal spermatogenesis with substantial levels of apoptosis, ultimately leading to male infertility.

### Progression of meiotic prophase is impaired in *Cfp1*^Stra8^ testes

**Fig. 2:**
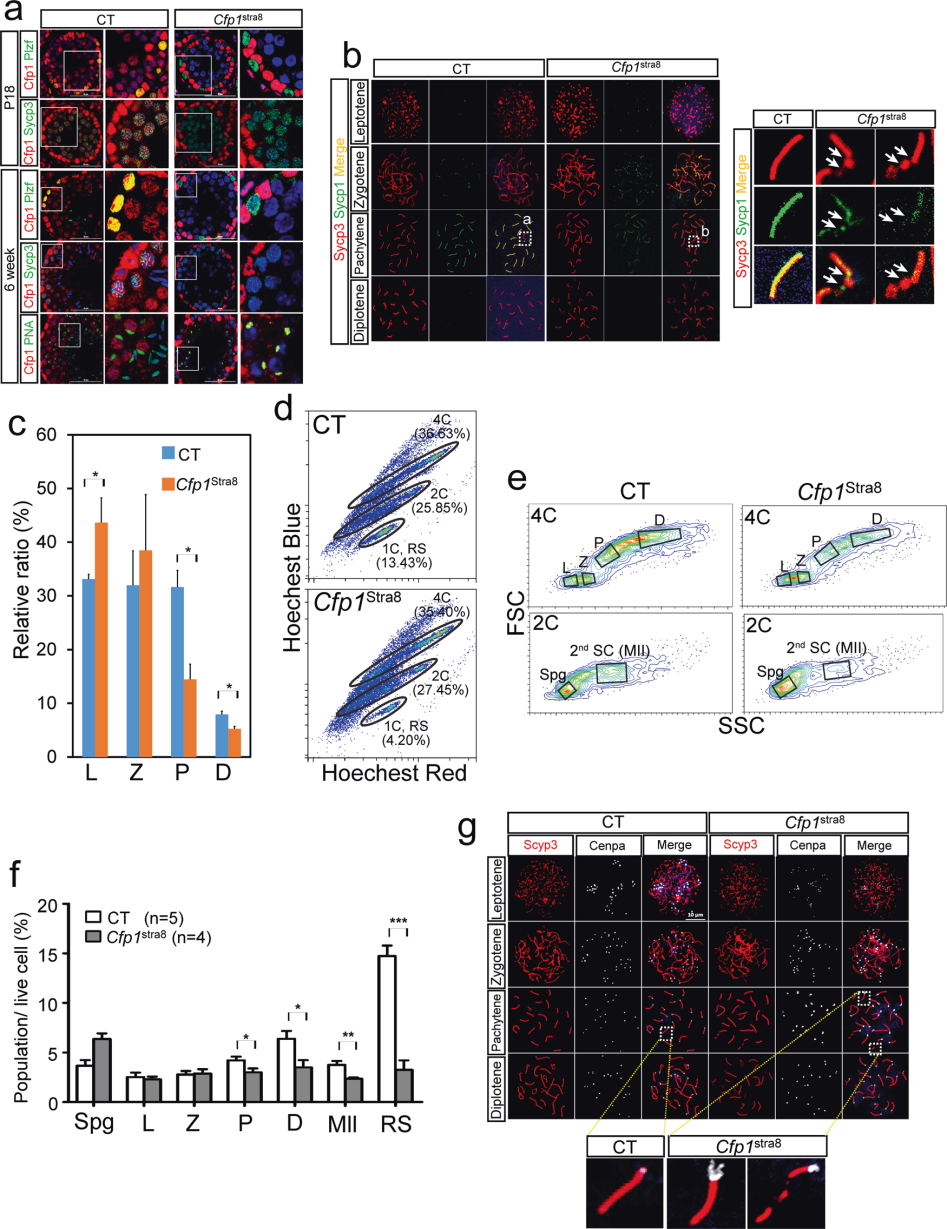
Cfp1 loss leads to meiotic arrest in *Cfp1*^*Stra8*^ spermatocytes. **a** Immunofluorescence with anti-Cfp1 (red), anti-Plzf (green, a spermatogonia marker), anti-Sycp3 (green, a spermatocyte marker), and PNA (green, a spermatid marker) in seminiferous tubules of control (CT) and *Cfp1*^*Stra8*^ testes at P18 and 6 weeks old. DNA was stained with DAPI (blue). **b** Arrows indicate dot-like stained PNA(+) *Cfp1*^*Stra8*^ spermatid chromosome spreading assay on spermatocytes of CT and *Cfp1*^*Stra8*^ mice at meiotic prophase I. Chromosomes were stained with anti-Sycp3 (red) and anti-Sycp1 (green) antibodies. White boxes define the field of enlarged images. Arrows indicate aberrant synapses in *Cfp1*^*Stra8*^ pachytene. **c** Quantification of prophase I substages in chromosome spread of CT (blue) and *Cfp1*^*Stra8*^ testes (orange) (*n* = 4/group). L, leptotene; Z, zygotene; P, pachytene; D, diplotene. *p value < 0.05. **d** Flow cytometry analysis of the spermatogenic population in 3 wks CT and *Cfp1*^Stra8^ testes using Hoechst 33342 dye (both blue and red laser). **e** Plots showing 4C, 2C, and 1C populations in CT and *Cfp1*^Stra8^. Measurement of 4C and 2C subpopulations by back-gating the germ cell population in D). L: leptotene, Z: zygotene, P: pachytene, D: diplotene, Spg: spermatogonia, 2nd SC (MII): secondary spermatocyte. **f** Quantification of germ cells in the flow cytometry analysis (Fig. 2d, e). *p value < 0.05; **p value < 0.01; ***p value < 0.001. **g** Representative images of chromosome spreads stained with anti-Sycp3 (red) and anti-Cenpa (white) antibodies in CT and *Cfp1*^*Stra8*^ spermatocytes. White boxes represent enlarged images.

Given that incomplete spermatogenesis causes cell apoptosis and sterility, we performed immunofluorescence and chromosome spread assays to examine the status of meiotic progression in *Cfp*1^Stra8^ seminiferous tubules (Fig. 2). Immunofluorescence revealed no obvious change in the number of Cfp1(+) cells in *Cfp1*^*Stra8*^ germ cells at P3 (data not shown). However, germ cells lacking Cfp1 began to appear in the tubules from P7, and the number of cells increased significantly at P18 compared to that in CT mice (data not shown). Our data are highly reminiscent of animal models involving defects in meiotic progression^37,38^. Then, we determined that defects occurred in *Cfp1*^*Stra8*^ testes at P18 and 6 weeks of age using stage-specific germ cell markers, including Plzf, Sycp3, and PNA for spermatogonia, spermatocytes, and spermatids, respectively (Fig. 2a). Immunofluorescence showed that Cfp1-negative spermatogonia and spermatocytes were present in *Cfp1*^*Stra8*^ seminiferous tubules at P18. However, no or few spermatids were detected in the 6-week-old *Cfp1*^*Stra8*^ seminiferous tubules (Fig. 2a). Interestingly, we found that PNA, which labels the acrosomal cap of spermatids and spermatozoa, exhibited a small dot-like pattern in *Cfp1*^*Stra8*^ spermatids, whereas a crescent-like pattern was observed in CT spermatids at 6 weeks of age, indicating unshaped morphology of round spermatids (Fig. 2a).

Next, using a chromosome spread assay, we found that *Cfp1*^*Stra8*^ spermatogonia could enter the first stage of the meiotic process but failed to form synapsed homologous chromosomes during pachynema. The relative number of normal synapsed chromosomes significantly decreased (Fig. 2b, c). Then, we employed flow cytometric analysis to directly measure the fraction of spermatogenic populations in cKO testes. One chromatid per chromosome (1C)-containing germ cell (round spermatid, RS) was overtly reduced in cKO testes (13.43% vs. 4.20%), whereas 4C (meiocytes in prophase I) and 2C [spermatogonia and 2nd spermatocytes (MII)] germ cells were not grossly changed in cKO testes (Fig. 2d). To further analyze the population of substaged germ cells in 2C and 4C, a back-gating strategy was used in the flow cytometric analysis. As shown in Fig. 2e, f, pachynema, diplonema, and MII were significantly diminished in the cKO testes. It is interesting to note that the number of spermatogonial cells was modestly increased in the cKO testes. We also found abnormal staining of centromere protein A (Cenpa) on synapsed chromosomes of *Cfp1*^*Stra8*^ spermatocytes, as they did not completely overlap at the phachytene stage (Fig. 2g).

### *Cfp1* loss causes aberrant expression of genes essential for meiosis

**Fig. 3:**
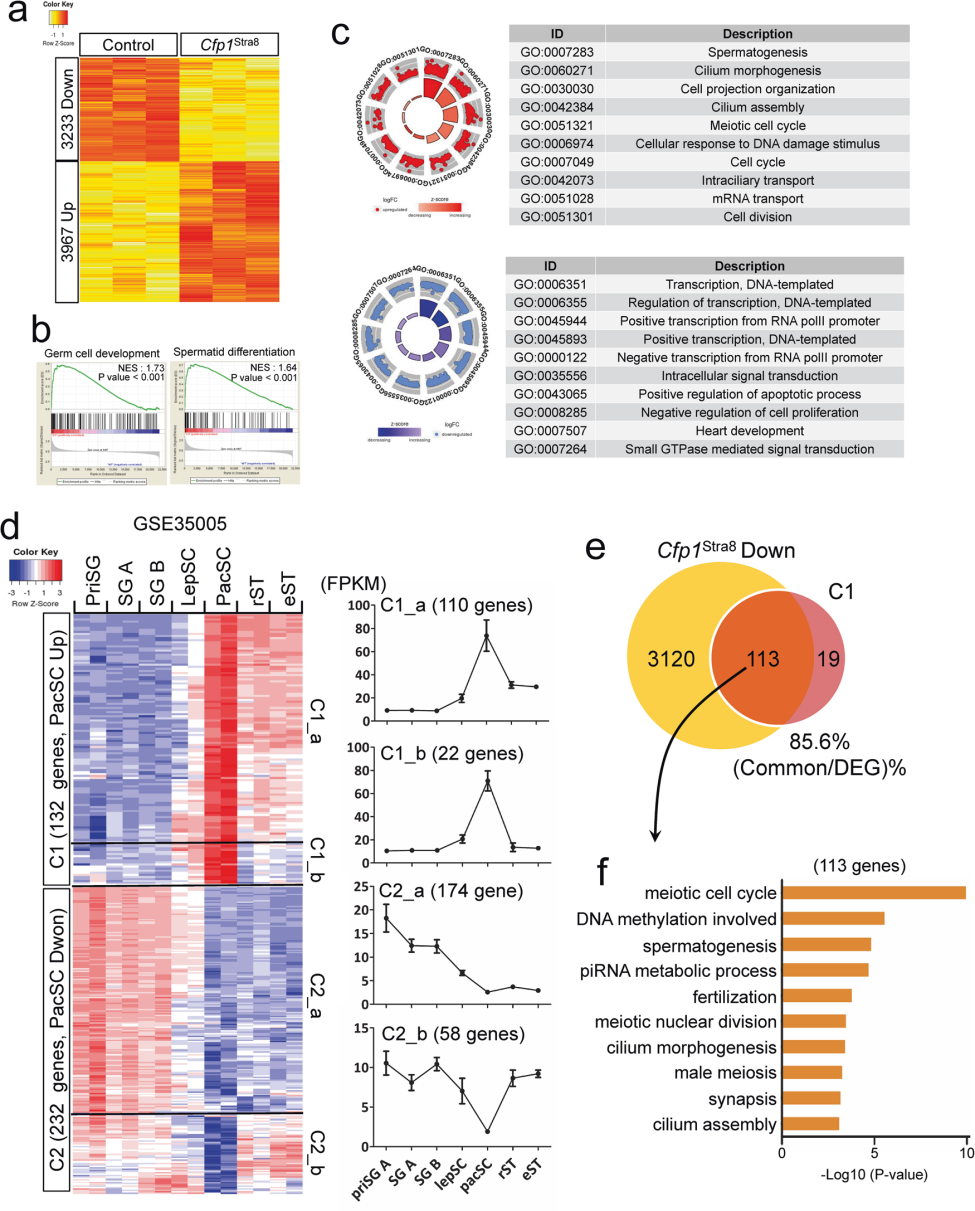
Aberrant gene expression in *Cfp1*^Stra8^ testes. **a** Heatmap showing differentially expressed genes (DEGs) between CT and *Cfp1*^Stra8^ P14 testes. Red and yellow indicate upregulated and downregulated genes, respectively. **b** Gene set enrichment analysis (GSEA) using the DEGs. NES; normalized enrichment score. **c** GOplot images showing GO terms with the DEGs. Each dot represents one gene in each GO term. Red and blue indicate downregulated and upregulated genes, respectively. **d** Heatmap showing DEGs during spermatogenesis. A publicly available RNA-Seq dataset (GSE35005) was used in the analysis. Note that 132 genes (C1) were upregulated, whereas 232 genes (C2) were downregulated in pachytene spermatocytes. Detailed subclassification of genes in C1 and C2. C1_a (110 genes), C1_b (22 genes), C2_a (174 genes), and C2_b (59 genes)] based on dynamic changes in their expression. priSG, primitive spermatogonia-A; SG-A, type A spermatogonia; SG-B, type B spermatogonia; LepSC, leptotene spermatocyte; PacSC, pachytene spermatocyte; rST, round spermatid; eST, elongated spermatid. **e** Venn diagram showing common genes between repressed genes in *Cfp1*^Stra8^ and C1 genes. Note that approximately 85% (113 out of 132) of genes upregulated at the pachytene stage (C1 genes) were repressed in *Cfp1*^Stra8^ testes. **f** GO term analysis with the 113 genes. Meiosis- and spermatogenesis-related GO terms were obtained with significant *p* values.

To explore the underlying mechanism of meiotic failure in *Cfp1*^Stra8^ spermatocytes, we performed microarray analysis in P14 testes (Fig. 3). As expected, our analysis revealed that *Cfp1* depletion perturbs gene expression, as 3233 genes were downregulated and 3967 genes were upregulated in cKO testes (Fig. 3a). The expression levels of some genes were confirmed using qRT–PCR analysis (Supplementary Fig. 2). As expected, the expression of numerous genes involved in meiosis was significantly reduced in *Cfp1*^Stra8^ testes, including *Sycp1*, *Sycp3*, *Syce1*, *Syce2*, and *Hormad1*. Interestingly, *Dmc1* gene expression was reversely increased. Gene set enrichment analysis (GSEA) revealed that among the 7200 DEGs, genes involved in germ cell development and spermatid differentiation were particularly enriched (Fig. 3b). We then analyzed the groups of genes found to be upregulated and downregulated using gene ontology (GO) term analysis and found that spermatogenesis was the top-ranked GO term among the repressed genes (Fig. 3c). In contrast, genes related to transcription were significantly upregulated in cKO testes.

We then aimed to identify key genes associated with failure in meiotic progression among the repressed genes. To that end, we reassessed a publicly available RNA-Seq dataset (GSE35005, Supplementary Fig. 3a) and obtained classified DEGs depending on their expression patterns during spermatogenesis (Fig. 3d). Our analysis revealed that 132 genes in C1 genes [persistently enhanced from the pachytene stage on (110 genes, C1_a) and only enhanced at the pachytene stage (22 genes, C1_b)] were upregulated at the pachytene stage of meiotic prophase I, whereas 232 genes in C2 genes [persistently repressed from the pachytene stage on (174 genes, C2_a) and only repressed at the pachytene stage (58 genes, C2_b)] were downregulated at the pachytene stage (Fig. 3d). Among the genes in the C1 genes, 113 out of 132 genes were repressed upon loss of Cfp1 (Fig. 3e) and were functionally associated with the meiotic cell cycle and spermatogenesis, as determined by GO term analysis (Fig. 3f). We also found that 129 out of 232 genes in the C2 genes were upregulated in *Cfp1*^Stra8^ testes (Supplementary Fig. 3b). However, no meiosis- or spermatogenesis-related GO terms were found among the common genes (Supplementary Fig. 3c). Our analysis further revealed that the expression of 21 out of 22 genes in C1_b was significantly repressed in *Cfp1*^Stra8^ testes (Supplementary Fig. 3d). Collectively, our microarray analysis demonstrates that Cfp1 is required for the temporal expression of genes that are indispensable for male meiotic progression.

### *Cfp1* in spermatocytes is enriched at transcription start sites of the pachynema-predominant genes

**Fig. 4:**
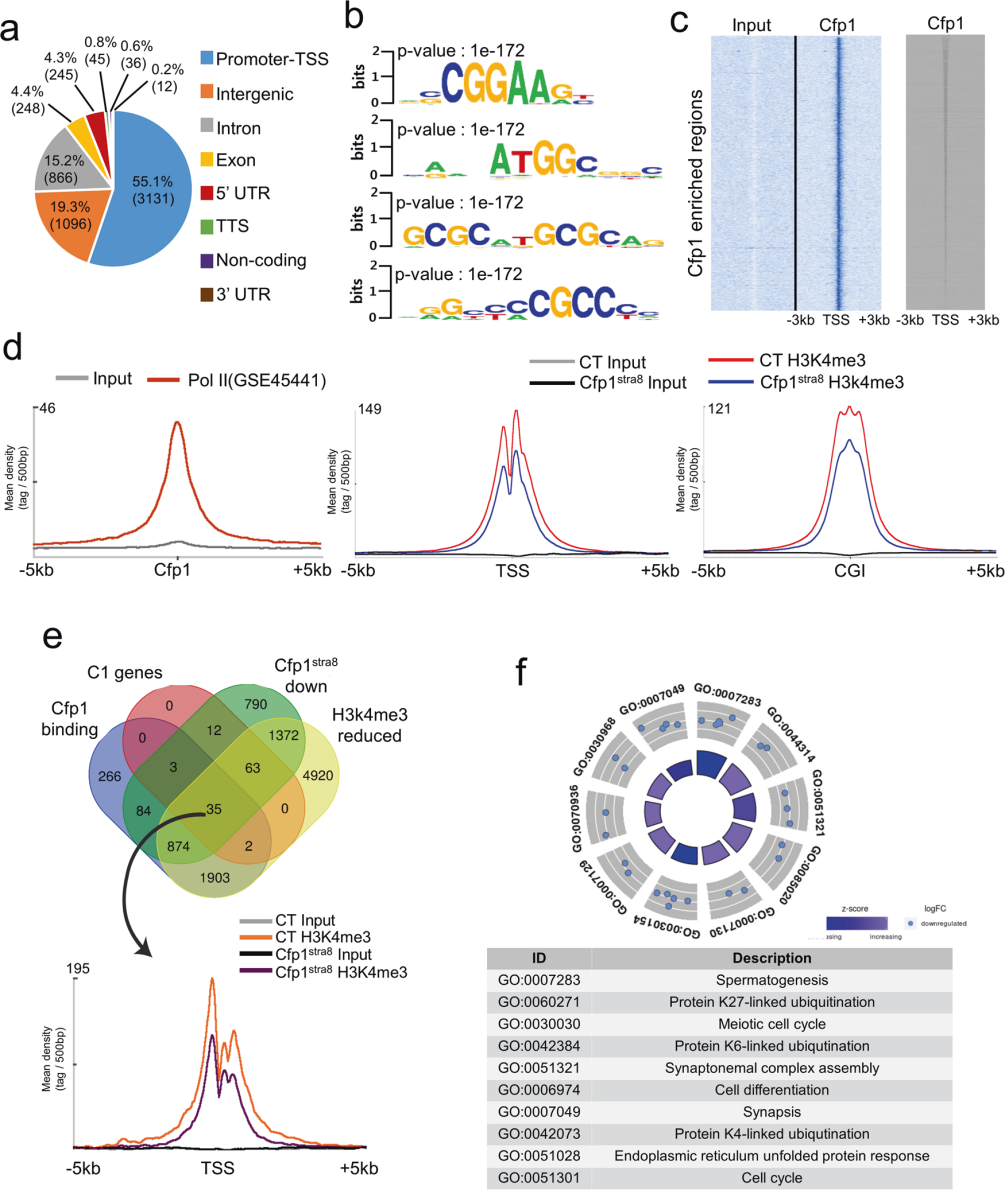
CFP1 is mainly enriched at the promoter/TSS in spermatocytes. **a** Pie chart showing CFP1 enrichment in spermatocytes genome-wide. Note that more than half of the Cfp1 enrichment was detected at the promoter/TSS. The rest included intergenic regions (19.3%), introns (15.2%), exons (4.4%), UTRs (4.5%), and other genomic regions. UTR, untranslated region. TTS, transcription termination site. Analysis of de novo Cfp1-binding motifs using ChIP-Seq data. **b** Binding motifs containing cytosine (C) and guanine (G) dinucleotides were identified. **c** Heatmaps showing Cfp1 enrichment at transcription start sites (TSSs) and CpG islands. **d** Enrichment of RNA polymerase II (PolII) occupancy (GSE45441) at Cfp1-binding sites and H3K4me3 levels at TSSs and CGIs in *Cfp1*^Stra8^ spermatocytes. **e** Venn diagram showing the numbers of overlapping genes among Cfp1-binding sites, C1 genes, genes downregulated in *Cfp1*^Stra8^ testes and genes with reduced H3K4me3 levels in *Cfp1*^Stra8^ spermatocytes. **f** Mean tag density of H3K4me3 ChIP-Seq in 35 common genes is presented. GO term analysis with 35 common genes is illustrated.

To examine the enrichment of Cfp1-binding genome-wide, ChIP-Seq was performed in spermatocytes isolated from P21 WT mice. As shown in Fig. 4a, more than half of the Cfp1-binding sites (55.1%) were found at promoter/transcription start sites (TSSs). The remaining instances of binding were identified at intergenic (19.3%), intron (15.2%), and other regions with <5% each. Consistent with our data, previous studies have also shown that Cfp1 is primarily enriched at the promoter/TSS in different cell types, such as ESCs and lymphocytes^26–29^. De novo binding motif analysis highlighted that Cfp1 preferentially binds to DNA sequences containing cytosine (C) and guanine (G) dinucleotides (Fig. 4b). Accordingly, Cfp1-binding sites overlapped with TSS, promoter CpG islands (CGIs) and RNA polymerase II (PolII) binding sites (GSE45441) (Fig. 4c, d). Next, we performed H3K4me3 ChIP-Seq analysis in cKO spermatocytes and found that H3K4me3 levels at TSS and CGI were diminished in cKO spermatocytes (Fig. 4d). Further analysis revealed that Cfp1 binds directly to the TSS of approximately 30% of the downregulated genes (996 out of 3233 genes) (Fig. 4e). Importantly, 35 Cfp1 target genes out of 132 C1 genes were identified as repressing both their expression and H3K4me3 levels at TSSs (Fig. 4e). GO term analysis revealed spermatogenesis and the meiotic cell cycle as the top-ranked biological pathways among the common genes (Fig. 4f).

### Activation of pachytene-specific genes is mediated by Cfp1-controlled H3K4me3 occupancy at TSSs

**Fig. 5:**
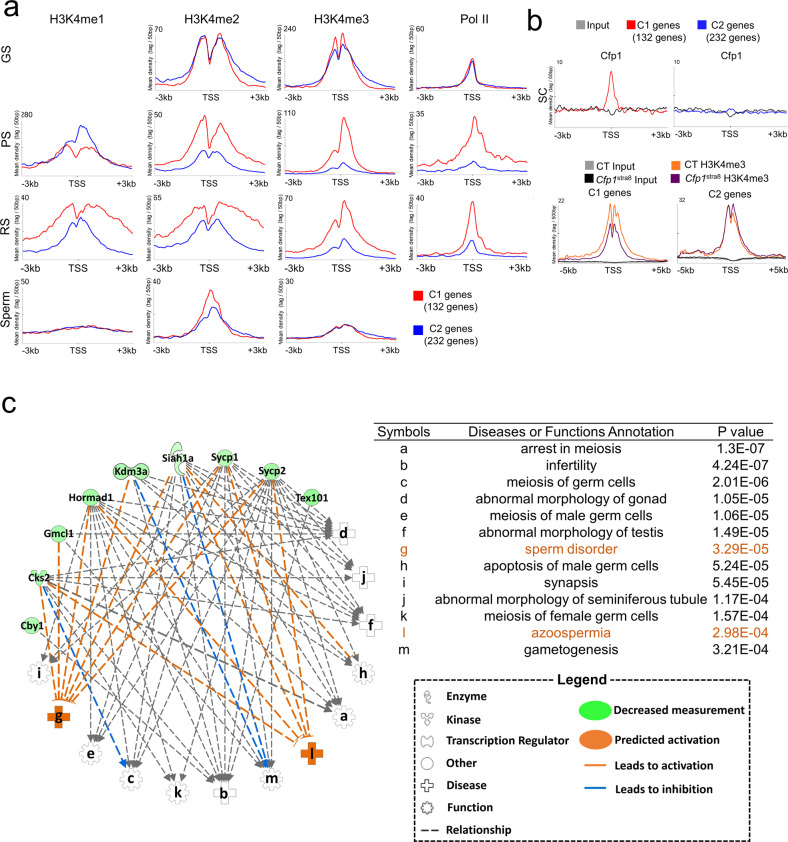
Cfp1 function is critical for transcriptional activation of genes upregulated at the pachytene stage. **a** Changes in H3K4me1/2/3 levels (GSE49624, GSE69946, GSE55471, GSE79227, and PRJNA281061) and PolII occupancy (GSE45441) in the C1 and C2 genes of germline stem cells (GS), spermatocytes at the pachytene stage (PS), round spermatids (RS) and sperm. Note that enrichment of H3K4me2/3 and PolII in C1 genes increased in PS and RS, whereas those in C2 genes decreased in the cell types. **b** Enrichment of Cfp1 binding (upper) and change of H3K4me3 levels in *Cfp1*^Stra8^ spermatocytes (lower) at TSS of genes in C1 and C2. SC, spermatocyte. **c** Ingenuity pathway analysis (IPA) of the 35 genes. Shapes and lines are color-coded based on predicted associations and functions as indicated in the legend box. The alphabet symbols represent associated disorders in reproduction with *p* values.

To investigate the mechanistic link between histone H3K4me and the activation of pachytene-specific genes, we reassessed publicly available H3K4me datasets (GSE49624, GSE69946, GSE55471, GSE79227, and PRJNA281061). The analysis revealed that the enrichment pattern of H3K4me2/3 in the C1 genes clearly diverged from that of the C2 genes at the pachytene stage. Notably, no differences in H3K4me2/3 and PolII (GSE69946 and GSE45441) enrichments between C1 genes and C2 genes were observed in germline stem cells (GSs), whereas there was more enrichment in the H3K4me2/3 and PolII in C1 genes over the C2 genes in pachytene-stage spermatocytes (PS) and round spermatids (RS) (Fig. 5a). Importantly, Cfp1 was clearly enriched in the C1 genes but not in the C2 genes of spermatocytes (SC) (Fig. 5b). Accordingly, the levels of H3K4me3 in the C1 genes, but not in the C2 genes, were decreased at TSS (Fig. 5b). Next, using the 35 common genes, we performed an Ingenuity Pathway Analysis (IPA) to infer possible relationships with human diseases. The networks with their respective scores obtained from IPA are shown in Fig. 5c. Our analysis identified 9 genes (*Cby1*, *Cks2*, *Gmcl1*, *Hormad1*, *Kdm3a*, *Siah1a*, *Sycp1*, *Sycp2*, and *Tex101*) that were associated with the top 13 most predicted categories of human diseases listed in Fig. 5c. Interestingly, the IPA predicted multiple human disorders related to reproductive failures, including sperm disorders and azoospermia, with highly significant *p* values (Fig. 5c). Other predicted dysfunctions in reproduction, such as infertility, abnormal morphology of male reproductive organs, spermatogenesis, meiotic arrest, and apoptosis in male germ cells, were also obtained. Our analysis indicates that dysfunction or deficiency of CFP1 in male germ cells is likely associated with reproductive defects in humans as well.

## Discussion

Meiosis is an essential step in spermatogenesis and represents an intriguing model system to study epigenetic regulation. Evidence suggests that aberrant epigenetic control in meiosis leads to infertility due to failure to bypass meiotic checkpoint arrest^39,40^.

In the present study, we show that Cfp1 plays an essential role in meiotic progression via regulation of transcription but is likely dispensable for DSB formation, as no co-occupancy of Cfp1 and DSB-associated factors (Prdm9, Spo11, and Dmc1) was detected (data not shown).

We revealed that Cfp1 is highly expressed in the testes, and its cellular level varied throughout the process of germ cell differentiation. Specifically, the Cfp1 level was highest in spermatogonia and gradually decreased in spermatocytes and spermatids (Supplementary Fig. 1). The infertility of *Cfp1*^Stra8^ mice was attributed to meiotic arrest, in which aberrant formation of lateral and central elements resulting in patchy staining of Sycp1 and Sycp3 was detected in a chromosome-spread assay. Indeed, our microarray analysis showed reduced expression of Sycp1 and Sycp3 in *Cfp1*^Stra8^ meiocytes. Our Cenpa staining suggests that the pachynemas showing incomplete centromere pairing were eliminated as their numbers at diplotene were reduced (Fig. 2g).

Given that Cfp1 is highly expressed in stem cells, including ESCs, HSCs, and SSCs, and the fact that differentiation defects occur because of *Cfp1* loss, it is feasible that Cfp1 function is required for regulating core genes that orchestrate lineage determination and differentiation processes. Our analysis also indicates that many DEGs are unlikely to be direct targets of Cfp1 but rather a consequence of meiotic failure in *Cfp1*^Stra8^ spermatocytes. Recently, using conditional KO approaches, two independent studies investigated the roles of Cfp1 in male germ cell development. Tian et al. reported a dispensable Cfp1 role in male fertility and germ cell meiosis^41^, whereas Jiang et al. claimed an essential Cfp1 role in male fertility and DSB repair and proper crossover formation^42^. Unlike the studies that only focus on DSB repair and crossover formation, our study reveals that Cfp1 function mechanistically links to transcriptional regulation of meiotic genes during spermatogenesis and its depletion causes sterility. Different cKO strategies might reconcile the different reproductive phenotypes in each cKO strain. Nevertheless, further analyses are needed to clarify what caused the difference.

Chromatin structure is dynamically regulated in meiocytes, and therefore, chromatin status is closely correlated with gene expression^43^. Consistent with this, there is enrichment of both H3K4me2/3 and PolII at the promoter/TSS, resulting in activation of gene transcription. One of the most prominent chromatin dynamics in spermatogenesis was observed during the mitosis-to-meiosis transition from spermatogonia to spermatocytes. Unlike somatic cells, H3K4me3 distribution and associated enzymes are unique in meiotic germ cells. H3K4me at TSSs is presumably mediated by Setd1a/b complexes, whereas those at DSBs are mediated by Prdm9. Prdm9 encodes a PR/SET domain with methyltransferase activity for H3K4me and H3K36me and a ZF domain with high sequence specificity for hotspots^7–10,44^. Although it is generally accepted that Prdm9 associates with DSBs, no positive correlation between de novo open chromatin formation and Prdm9 binding was revealed at the pachynema stage^14^.

Mice deficient in Setd1a or Setd1b exhibit embryonic lethality with a reduction in H3K4me3 genome-wide and global changes in gene expression^45^. Similarly, Cfp1 depletion alters gene expression in testes. Our genome-wide analysis showed that Cfp1 is most frequently associated with promoter/TSS regions, as observed in other cell types, and enrichment of Cfp1 and H3K4me3 levels are closely associated with the activation of stage-specific genes (Fig. 5). Furthermore, the binding motifs of Cfp1 in spermatocytes are quite similar to those in other cell types but differ from those of Prdm9 in spermatocytes^31,46,47^.

One important question that has yet to be addressed is how H3K4me3 at DSBs is differentially regulated from that which occurs at TSSs. Although in vitro studies have indicated that Prdm9 can interact with Cfp1, it is unclear whether this interaction occurs in vivo. Our combined analysis of Cfp1 and Prdm9 ChIP-Seq data fails to support a role for Cfp1 in DSB formation in meiocytes, as no Cfp1 peak corresponding to Prdm9 binding was found (data not shown). Consistently, no change in Dmc1 occupancy or enrichment was found in cKO spermatocytes (data not shown).

Therefore, our observations are in agreement with previous studies that report preferential binding of Cfp1 to its targets (H3K4-enriched nonmethylated CGIs) via both CXXC and PHD domains^22,24^. There have yet to be any reports that CGIs exist in close proximity to recombination hotspots in mice.

Collectively, our study suggests that Cfp1 is essential for meiotic gene expression by depositing H3K4me at the promoter of target genes. Without the guidance of Setd1 by Cfp1, the sensing of nonmethylated CpG and H3K4me on the targets is impaired, thus leading to abnormal spermatogenesis. We have highlighted the role of Cfp1 as a guardian of the meiotic process during spermatogenesis by orchestrating spermatogenesis-related factors. Given that spermatogenesis is a complex process, there are a myriad of factors that must be tightly regulated to ensure efficient production of mature spermatozoa. These data will help to gain a better understanding of the complex cellular process and shed light on the contribution of genetic factors to male idiopathic azoospermia.

## Supplementary information

suppl_R2
