# Supplementary material for: Epigenetic regulator Cfp1 safeguards male meiotic progression by regulating meiotic gene expression
Source: Exp Mol Med. 2022 Aug 2;54(8):1098–108. doi: 10.1038/s12276-022-00813-0 (PMC9440128; doi:10.1038/s12276-022-00813-0)
Supplement: Supplementary file 1 — suppl_R2 [file 12276_2022_813_MOESM1_ESM.pdf]

Supplementary Information

Epigenetic regulator Cfp1 safeguards male meiotic progression by regulating meiotic gene expression

Byeong Seong Ki[1]*, Sung Han Shim[1]*, Chanhyeok Park[2]*, Hyunjin Yoo[2]*, Hyeonwoo La[2], Ok-Hee Lee[1], Youngjoo Kwon[3], David G. Skalnik[4], Yuki Okada[5], Ho-Geun Yoon[6], Jin-Hoi Kim[2], Kwonho Hong[2], Youngsok Choi[2]

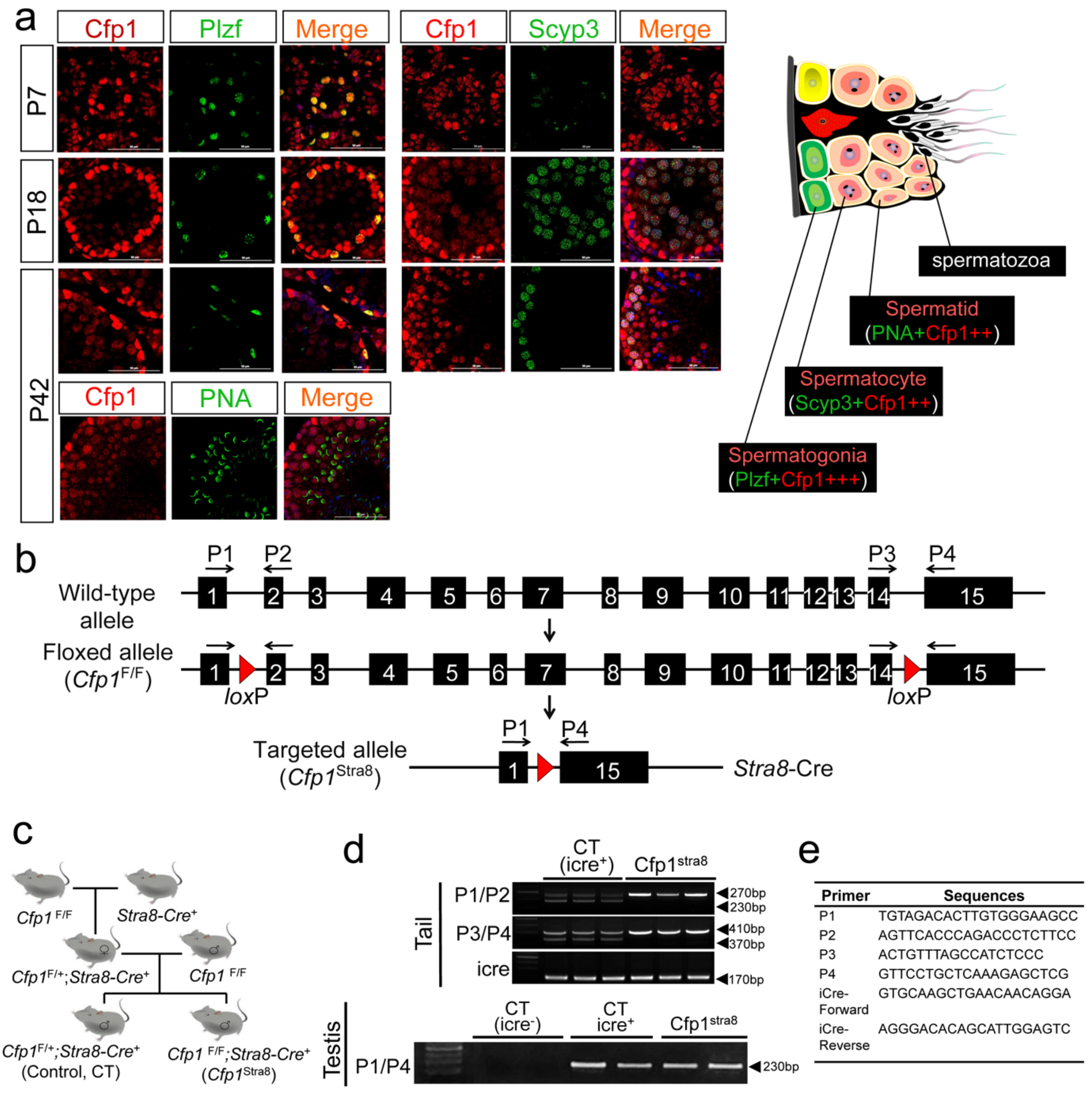

Supplementary Fig. 1 Spatiotemporal expression of *Cfp1* in the testes and generation of mice lacking *Cfp1* in male germ cells. a Immunofluorescence with anti-Cfp1 (red color) and germ cell markers [anti-Plzf (spermatogonia), anti-Sycp3 (spermatocyte) and PNA (spermatid), green color] antibodies in testes at various developmental stages (postnatal day 7 (P7), P18 and P42). DNA was stained with DAPI (Blue). Representative images from at least three independent experiments were obtained. * indicates lumen of seminiferous tubule. + in

cartoon indicates relative intensity of Cfp1 staining. Scale bars; 50μm. **b** Each black box represents one exon of mouse *Cfp1*. Arrows indicate the location of primers (P1~ P4) used for genotyping. Red triangles indicate two *lox*P sites. Exons 2-14 of *Cfp1* floxed by *lox*P sequences (*Cfp1*$^{F/F}$) were designed to be excised by Tg(*Stra8*-icre) line (*Cfp1*^{Stra8}). **c** Illustrations of breeding scheme to generate both CT (*Cfp1*$^{F/+}$;*Stra8*-icre(+)) and cKO mice (*Cfp1*$^{F/F}$;*Stra8*-icre(+), *Cfp1*^{Stra8}). **d** PCR genotyping to confirm the cKO mouse. The PCR primers, P1, P2, P3, and P4, were used to identify heterozygotes or homozygotes for the *Cfp1* cKO allele using tail tissue. With P1 and P2 primer set, CT shows two bands (270 bp and 230 bp), whereas *Cfp1*^{Stra8} contains only upper band (270 bp). With P3 and P4 primer set, CT shows two bands (410 bp and 370 bp), whereas *Cfp1*^{Stra8} contains only upper band (410 bp). Cre band (170 bp) was detected with icre primers. In testis tissues, P1 and P4 primers were used to identify Cfp1 deletion after Cre recombination (230 bp). **e** Primer sequence information for genotyping.

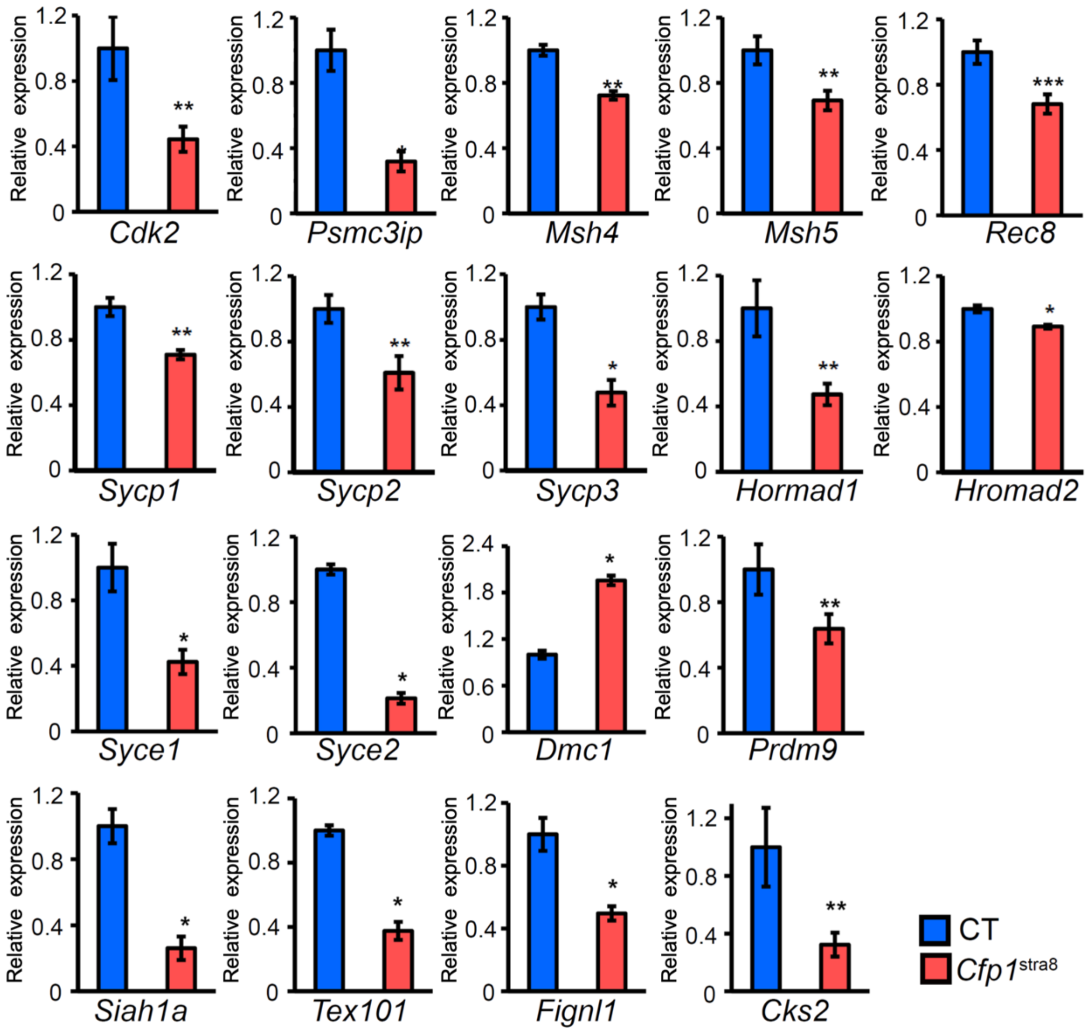

Supplementary Fig. 2 Confirmation of gene expression obtained from microarray analysis. Bar graphs of qRT-PCR analysis show gene expression changes of selected genes in isolated P14 spermatocytes of control (CT) and *Cfp1*^{Strа8}. * and ** represent genes displaying statistically significant changes in their expressions.

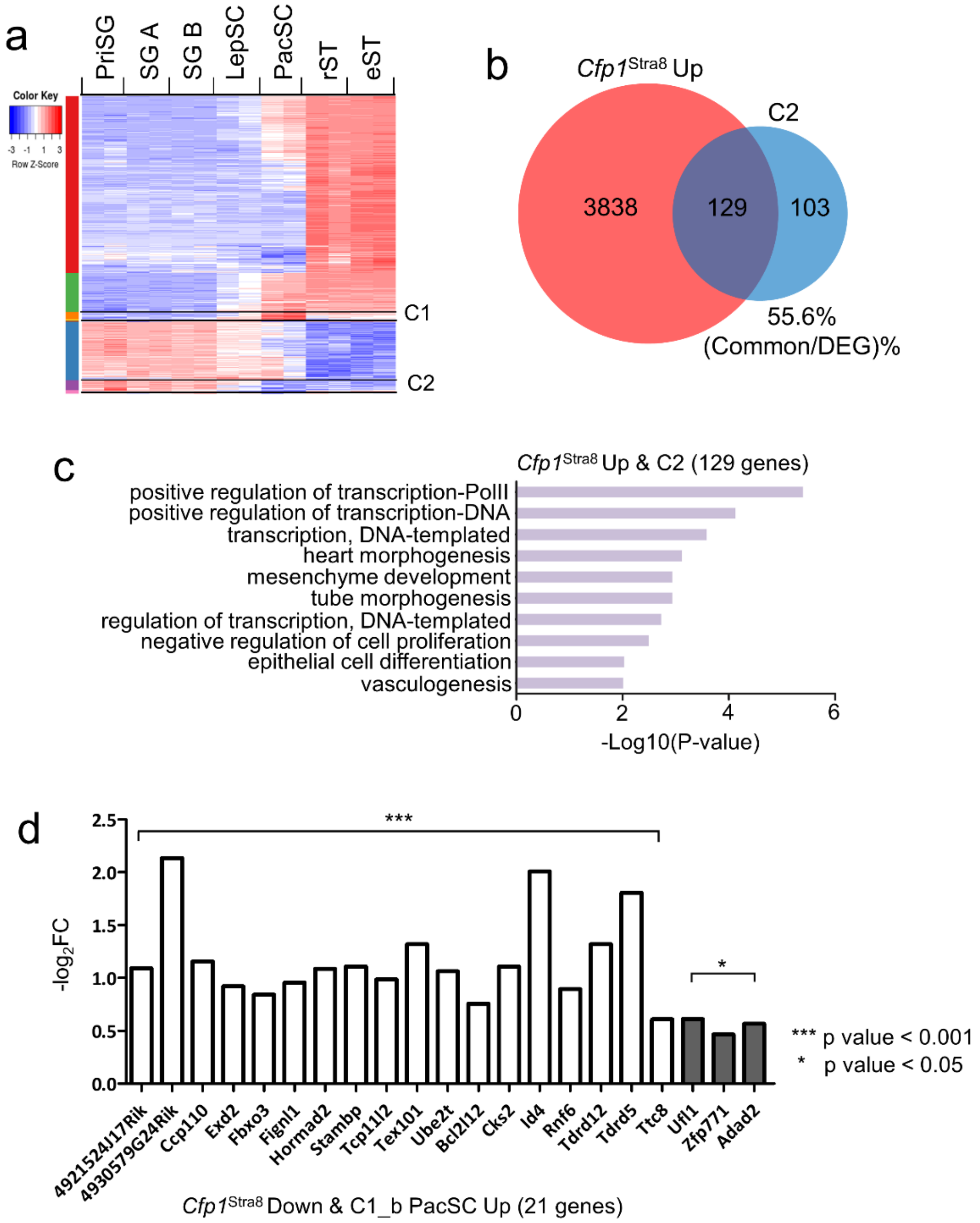

Supplementary Fig. 3 Assessment of gene expression pattern during spermatogenesis. a Heatmap showing dynamic change of gene transcription during spermatogenesis. priSG; primitive spermatogonia-A, SG-A; type A spermatogonia, SG-B; type B spermatogonia, LepSC; leptotene spermatocyte, PacSC; pachytene spermatocyte, rST; round spermatid, eST; elongated spermatid. Genes used in the main Fig. 4D are marked as C1 and C2. **b** Venn diagram showing number of overlapping genes (129 genes) between genes up-regulated in *Cfp1*$^{\mathrm{Stra8}}$ testes and genes in C2 cluster. **c** Bar graph showing GO terms analyzed with the 129 genes. **d** Bar graph showing -log$_2$FC values of 21 genes identified as overlapping genes between genes down-regulated in *Cfp1*$^{\mathrm{Stra8}}$ testes and genes in C1_b cluster. FC; fold change. *; $p<0.05$, ***; $p<0.001$.

Supplementary Table 1. Primer sequences.

Gene symbol		Primer sequences	product size (bp)
Cdk2	forward	CCTGCTTATCAATGCAGAGGG	214
	reverse	GTGCTGGGTACACACTAGGTG	
Psmc3ip (Hop2)	forward	CCCAGGACGTGTTCGGAAAC	158
	reverse	CACTGTGTCAAACTGGTTCTGAT	
Rec8	forward	TATGTGCTGGTAAGAGTGCAAC	133
	reverse	TGTCTTCCACAAGGTACTGGC	
Syce1	forward	CAGCCGTTGGGTATGGAGC	131
	reverse	CCCACTTTCTGCAACTTTTTCAC	
Syce2	forward	TGGACTCTAGCATTGAAACCCT	193
	reverse	TCCTGAATGATTTTGCTGTGGT	
Tex101	forward	TTTGCCAAGAAACGGTCCTG	238
	reverse	CCTGACATGTTGGAGGTTGC	
Dmc1	forward	CCCTCTGTGTGACAGCTCAAC	114
	reverse	GGTCAGCAATGTCCCGAAG	
Msh4	forward	TCCAGGCCGAGTACGGAAG	153
	reverse	TGAGCTTGTACCAACAGGAAAAC	
Msh5	forward	CCTGGGCATTGCTTACTATGAC	122
	reverse	CAACAGACTGGGGGTTGATTT	
Prdm9	forward	CTGAATACAAGTGGCTCAGAACA	160
	reverse	CCTCATAGGCAAGGCCCTTTC	
Sycp1	forward	CAAAAGCCCTTCACACTGTTCG	162
	reverse	GTTTTCCCGACTGGACATTGTAA	
Sycp2	forward	GACACTGAAACCGAATGTGGA	165
	reverse	TGTGGGTCTTGGTTGTCCTTT	
Sycp3	forward	AGCCAGTAACCAGAAAATTGAGC	106
	reverse	CCACTGCTGCAACACATTCAT	
Hormad1	forward	GGCTCCTAGCTGTTTCAGTATCT	153
	reverse	GCATCCACTTCACTAGCTGTG	
Hormad2	forward	CCGGGAAGACAAAAAGTGTCC	203
	reverse	TGCTGCTGTCAAAGTCCATAG	
Siah1a	forward	TGTTTGTAGCAACTGTCGCC	159
	reverse	TCGGTGTGTGGCAGAGTTAT	
Fignl1	forward	TGTGCAGGTGGACGAATGG	158
	reverse	TGAACAGGTTGGTAGCAAAGAC	
Cks2	forward	TCGATGAGCACTACGAGTACC	159
	reverse	TATGCGGTTCTGGCTCATGA	

Supplementary Table 2. List and information of primary antibodies.

Primary antibody	Host species	Dilution	Product
Plzf	mouse	1:300	Santa Cruz sc28319
Sycp3	mouse	1:400	Abcam ab97672
Sycp1	rabbit	1:400	Abcam ab15090
Gata-4	rabbit	1:400	Abcam ab84593
H3K4me1	rabbit	1:500	Abcam ab8895
H3K4me2	rabbit	1:500	Abcam ab7766
H3K4me3	rabbit	1:500	Abcam ab8580
H3K4me3 (ChIP-seq)	rabbit	3ul/IP	Active Motif 39159
Cenp-A	rabbit	1:500	Cellsignal c51a7
Cfp1 (IF)	rabbit	1:200	Sigma-Aldrich HPA044511
Cfp1 (ChIP-Seq)	rabbit	15ug/IP	Bethyl lab A303-161A
